# Analysis of Ocular Surface Characteristics and Incidence of Dry Eye Disease in Systemic Lupus Erythematosus Patients Without Secondary Sjögren's Syndrome

**DOI:** 10.3389/fmed.2022.833995

**Published:** 2022-03-07

**Authors:** Zhengyu Gu, Qinyi Lu, Ao Zhang, Zong Wen Shuai, Rongfeng Liao

**Affiliations:** ^1^Department of Ophthalmology, Anhui Medical University, Hefei, China; ^2^Departments of Rheumatology and Immunology, Anhui Medical University, Hefei, China

**Keywords:** dry eye disease, tear film, ocular surface, Meibomian gland, Systemic Lupus Erythematosus

## Abstract

**Objective:**

To investigate the differences in ocular surface characteristics, tear film quality, and the incidence of dry eye disease (DED) between Systemic Lupus Erythematosus (SLE) patients and healthy populations.

**Methods:**

This age and gender-matched cross-sectional study included 96 SLE patients without secondary Sjögren's syndrome (SS) and 72 healthy subjects. The Ocular Surface Disease Index (OSDI), tear meniscus height (TMH), non-invasive tear film breakup time (NIKBUT), meibography, and tear film lipid layer grade were assessed. A receiver operative characteristic (ROC) curve was constructed to evaluate the predictive value of risk factors.

**Results:**

Compared with the control subjects, a significantly greater proportion of SLE patients met the TFOS DEWS II DED diagnostic criteria (34.3 vs. 18.1%, *P* = 0.019). SLE patients without SS had higher OSDI scores [10.0 (4.5,18.0) vs. 5.0 (2.5,11.9), *P* < 0.001], and shorter NIKBUT [9.6 (6.6,15.0) vs. 12.3 (8.4, 15.8), *P* = 0.035]. Furthermore, TMH, Tear film lipid layer grade, and Meibomian gland (MG) dropout in SLE patients were worse than those in control subjects (all *P* < 0.05). For ROC analysis, the area under curve (AUC), sensitivity and specificity of prediction were 0.915, 75.8 and 92.1% for the combination of SLE disease activity index (SLEDAI), age and NIKBUT.

**Conclusions:**

SLE patients without SS exhibited a higher risk for DED than healthy subjects, and the poorer Meibomian gland function in SLE patients may potentially contribute to the development of DED. The combined parameters of SLEDAI, age and NIKBUT showed a high efficiency for the diagnosis of DED in SLE patients, with practical clinical applications.

## Introduction

Dry eye disease (DED) is a multifactorial disease of the ocular surface, which is characterized by the loss of tear film homeostasis accompanied by ocular surface symptoms (1, 2). The etiology includes tear film instability, hyperosmolarity, ocular surface inflammation, and neurosensory abnormalities (3, 4). In 2017, The Tear Film and Ocular Surface Society Dry Eye Workshop II (TFOS DEWS II) redefined DED and emphasized the need for further research examining the associations of the DED with autoimmune disease and noted that autoimmune disease was a high risk factor for DED. There is an association between aqueous-deficient DED and autoimmune diseases, especially Sjögren's syndrome (SS), which is characterized by chronic inflammation of the salivary and lacrimal glands (5).

SS can be classified in two types: primary SS (pSS) and secondary SS (sSS), in which SS can occur concomitantly with other autoimmune diseases, the most common being rheumatoid arthritis (RA), systemic lupus erythematosus (SLE) or scleroderma. Many studies have confirmed that patients with SS are prone to DED (6–8). The persistence of ocular surface inflammation plays an important role in the decline of tear film homeostasis in SS patients (9). SLE is a chronic autoimmune disease, which can affect multiple organs of the body (10). In SLE patients with SS, DED is a common complication (11). In SLE patients without SS, however, there have been few studies on the incidence and the etiological subcategories of DED. In addition, whether the SLE activity is a risk factor for DED is unclear.

The TFOS DEWS II Diagnostic Methodology Report proposed a more appropriate examination sequence and technique to guide the clinical diagnosis of DED, and recommends non-invasive examination (12). The Keratograph 5M is a non-invasive examination instrument that can compensate for the shortcomings of the traditional examinations, and provides a non-invasive, reproducible and more comprehensive screening method for DED (13).

The purpose of this cross-sectional study was to investigate the differences in ocular surface characteristics, tear film quality, and the incidence of DED between SLE patients and healthy populations according to the diagnostic criteria and methodology recommended by the TFOS DEWS II Diagnostic Methodology Report. Furthermore, the potential predictors of DED among SLE patients were explored.

## Methods

### Subjects

This age and gender-matched cross-sectional study recruited 96 SLE patients without SS (96 eyes) and 72 healthy subjects (72 eyes) from the First Affiliated Hospital of Anhui Medical University. Right eye of each participant was selected for statistical purposes. If only one eye was diagnosed as DED, that eye was selected for statistical analysis. The study followed the tenets of the Declaration of Helsinki and was approved by the institutional Research Ethics Board. Informed consent was obtained from all subjects prior to any procedure.

Inclusion criteria: the initial diagnosis was consistent with the criteria for SLE established by the American Rheumatology Association (ACR) (14). Exclusion criteria: (1) Secondary Sjögren's syndromes (sSS) was ruled out according to the Classification criteria for Sjögren's syndrome proposed by the American-European Consensus Group (15); (2) other autoimmune diseases; (3) anterior segment ocular disease (except DED); (4) history of ocular surgery; (5) using topical eye drops within 1 weeks before the examination; (6) contact lens wear within 72 h.

The DED evaluation was carried out in accordance with the diagnostic criteria and methodology recommended by the TFOS DEWS II Diagnostic Methodology Report (12). The symptoms of DED, tear film parameters, and ocular surface characteristics of each participant were evaluated.

### Evaluation of Dry Eye Symptoms

Subjective symptoms of DED were assessed using the Ocular Surface Disease Index (OSDI) questionnaire which is the most widely used questionnaire for DED clinical trials (16). The 12-item OSDI includes three subscales. The score for each question ranges from 0 to 4. The total score ranges from 0 to 100, with higher score indicating the more severe dry eye symptomology.

### Ocular Surface Assessment

The Keratograph 5M instrument (OCULUS, Optikgeräte GmbH, Wetzlar, Germany) was used to noninvasively assess the ocular surface and tear film parameters, including Tear meniscus height (TMH), non-invasive tear film breakup time (NIKBUT), tear film lipid layer grade and meibography. The structure of Keratograph 5M is based on the principle of a placido ring to provide high-definition video with multi-wavelength light source for data analysis.

The infrared tear meniscus image was photographed with a Keratograph 5M instrument, and the TMH directly below the center of the pupil was measured with the inbuilt digital calipers. The average of the three measurements near the center of the meniscus was taken. Through the built-in software on Keratograph 5M, NIKBUT was automatically recorded as the time for the first distortion in the grid reflection to be detected. The average of three consecutive NIKBUT values was calculated in each case.

The tear film lipid layer was graded according to the Guillon-Keeler grading system: grade 0, non-continuous layer; grade 1, open meshwork; grade 2, closed meshwork; grade 3, wave/flow; grade 4, amorphous; and grade 5, colored fringes (17).

**Figure 1:**
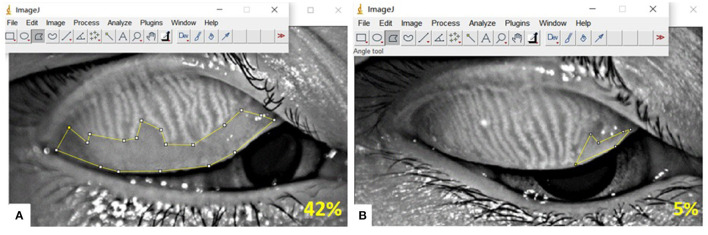
Detection of Meibomian gland (MG) dropout by Keratograph 5M, and MG dropout area was quantitatively measured using Image J software. **(A)** Infrared meibography of a participant, showing 42% MG dropout; **(B)** Infrared meibography of a participant, showing 5% MG dropout.

The meibomian gland (MG) dropout area was quantitatively measured using Image J software (http://imagej.nih.gov/ij) (Figure 1), and graded according to the five-point Meiboscale: Degree 0: no partial glands; Degree 1: ≤25% partial glands; Degree 2: >26 and ≤50% partial glands; Degree 3: >51 and ≤75% partial glands; Degree 4: >75% partial glands (18). Eyelid margin abnormalities, including lid margin notching, telangiectasia, and plugging were assessed (19).

The diagnostic criteria for DED and subtypes of DED were adapted from the rapid non-invasive dry eye evaluation algorithm, which has been previously validated and proven to have high diagnostic consistency with diagnostic criteria and methodology recommended by the TFOS DEWS II Diagnostic Methodology Report (20, 21). Patients with OSDI ≥ 13 and NIKBUT < 10 seconds were enrolled in the DED group. DED can be divided into three etiological subtypes: aqueous tear-deficient dry eye (criteria: diagnosis of DED and TMH < 0.2 mm), evaporative dry eye (criteria: diagnosis of DED and meibography grade >1 or tear film lipid layer grade ≤3) and mixed dry eye.

### SLE Disease Activity Evaluation

The SLE disease activity index (SLEDAI) was used to evaluate the SLE activity: inactivity: 0–4 points; mild activity: 5–9 points; moderate activity: 10–14 points; severe activity: ≥15 points (22).

### Statistical Analysis

SPSS 26.0 software was used for data analysis. Continuous and normally distributed variables were presented as means and standard deviation (SD), and an independent sample *t* test was used to assess group differences. No-normally distributed data was described by median (25% interquartile, 75% interquartile) and the Mann–Whitney *U* test was used to assess group differences. The chi-square test was used to assess group differences for categorical variables. Analysis of variance was used to compare the normally distributed data between the three groups, and the Kruskal–Wallis test was used to compare the no-normally distributed data between the three groups. The receiver operative characteristic (ROC) curves were plotted to evaluate the predictive value of risk factors. When *P* < 0.05 (bilateral), the difference was considered statistically significant.

## Results

In this age and gender-matched cross-sectional study, a total of 168 participants were included (96 SLE patients and 72 healthy subjects). The demographic and ocular surface characteristics of the participants are presented in Table 1.

**Table 1:** Demographic and ocular characteristic of SLE patients and healthy subjects.

**Parameters**	**SLE Patients (*n* = 96)**	**Healthy subjects (*n =* 72)**	***P***
**Demographics**			
Age, y	35.8 ± 10.9	38.4 ± 9.78	0.089
Female, *n*(%)	91 (94.7%)	67 (93.1%)	
**Dry eye symptom**			
OSDI (out of 100)	10.0 (4.5,18.0)	5.0 (2.5,11.9)	**<0.001**
**Tear film characteristics**			
NIKBUT(s)	9.6 (6.6,15.0)	12.3 (8.4, 15.8)	**0.035**
Tear film lipid layer grade (out of 5)	2 (2,3)	3 (2,3)	**0.030**
TMH (mm)	0.19 (0.16,0.23)	0.21 (0.17, 0.28)	**0.049**
**Eyelid characteristics**			
Meiboscale of Pult score (out of 4)	2 (1,3)	1 (1,2)	**0.010**
Eyelid margin notching	20 (20.8%)	11 (15.3%)	0.358
Eyelid margin telangiectasia	13 (13.5%)	2 (2.8%)	**0.015**
Eyelid margin plugging	21 (21.9%)	15 (20.8%)	0.871
**TFOS DEWS II DED diagnosis**	33 (34.3%)	13 (18.1%)	**0.019**
**Sub-categories of DED**			
Total DED	33	13	
ADDE	10 (30.3%)	1 (7.7%)	
EDE	9 (27.3%)	8 (61.5%)	
Mixed DED	14 (42.4%)	4 (30.8%)	

*Data is presented as mean ± SD, median and interquartile range (IQR, 25–75%), or number of participants (% of participants). SLE, Systemic Lupus Erythematosus; OSDI, Ocular Surface Disease Index; TMH, Tear Meniscus Height; NIKBUT, Non-invasive Tear Film Breakup Time. The bold values means significant results*.

### Dry Eye Symptoms and Ocular Surface Characteristics

**Figure 2:**
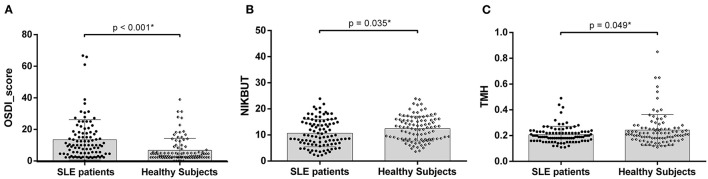
**(A)** Comparison of Ocular Surface Disease Index (OSDI) scores between the SLE patients and healthy subjects; **(B)** Comparison of non-invasive tear breakup time (NIKBUT) between two group; **(C)** Comparison of TMH, tear meniscus height between two group; Asterisks denote statistically significant difference (*P* < 0.05).

Compared with the healthy subjects, a significantly greater proportion of SLE patients met the TFOS DEWS II DED diagnostic criteria (34.3 vs. 18.1%, *P* = 0.019). OSDI scores were higher in SLE patients than in healthy subjects [10.0 (4.5,18.0) versus 5.0 (2.5,11.9), *P* < 0.001, Figure 2A]. SLE patients displayed shorter NIKBUT [9.6 (6.6,15.0) vs. 12.3 (8.4, 15.8), *P* = 0.035, Figure 2B] and lower TMH [0.19 (0.16,0.23) vs. 0.21 (0.17, 0.28), *P* = 0.049, Figure 2C].

**Figure 3:**
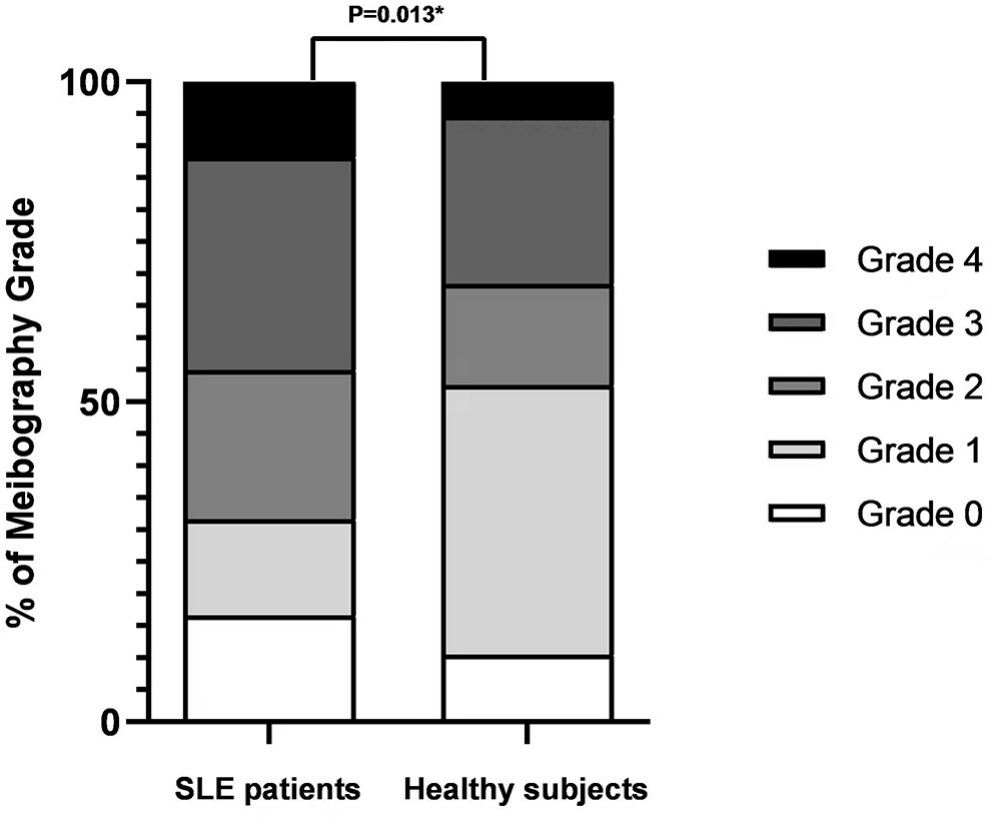
Meiboscale of Pult score distribution for SLE patients and healthy subjects. Bars represent the percentage of participants within each meibography grade. Asterisks denote statistically significant difference (*P* < 0.05).

According to the five-point Meiboscale, greater levels of MG dropout were observed in SLE patients than in healthy subjects [2(1,3) vs. 1(1,2), *P* = 0.013, Figure 3]. Compared with the healthy subjects, SLE patients had poorer tear film lipid layer grades [2(2,3) vs. 3(2,3), *P* = 0.030]. There were no significant differences in eyelid margin notching and plugging between SLE patients and healthy subjects (all *P* > 0.05).

### Classification of Subcategories of DED in SLE Group and Healthy Group

**Figure 4:**
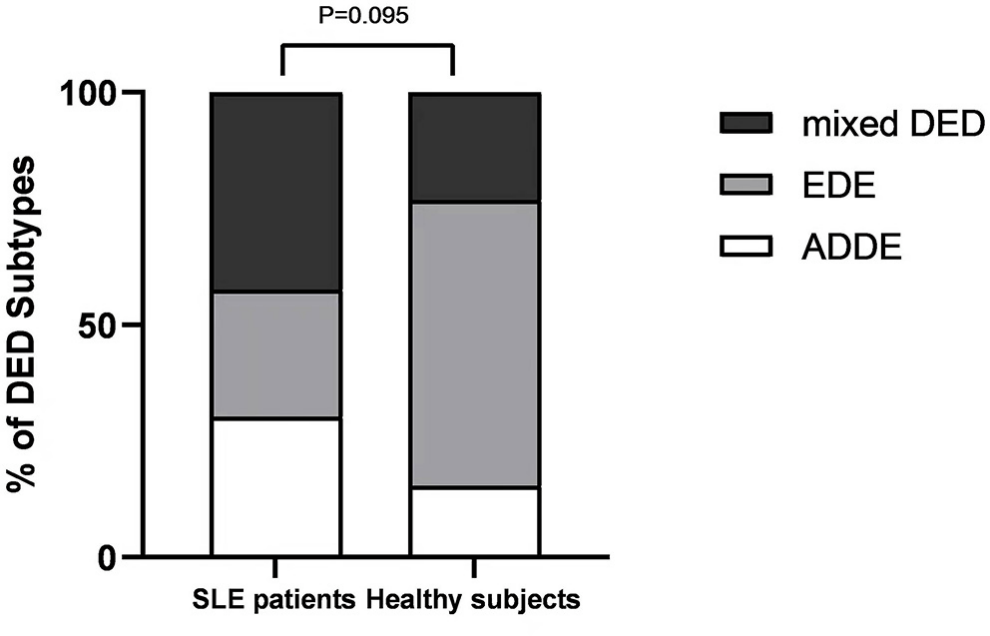
DED Subtypes distribution for SLE patients and healthy subjects. Bars represent the percentage of participants within each DED subtype.

According to the classification of TFOS DEWS II criteria, among the DED in SLE patients, there were 10 cases of aqueous tear-deficient dry eye (ADDE), 9 cases of evaporative dry eye (EDE), and 14 cases of mixed DED. In healthy subjects, there were 1 case of ADDE, 8 cases of DED, and 4 cases of mixed DED (Table 1). There was no significant difference between two group (*P* = 0.071) (Figure 4). In addition, there were no significant differences in age, sex, OSDI, SELDAI, and SLE course among the three DED subtypes of SLE patients.

### Correlation Between DED Symptoms and Signs in SLE Patients

**Figure 5:**
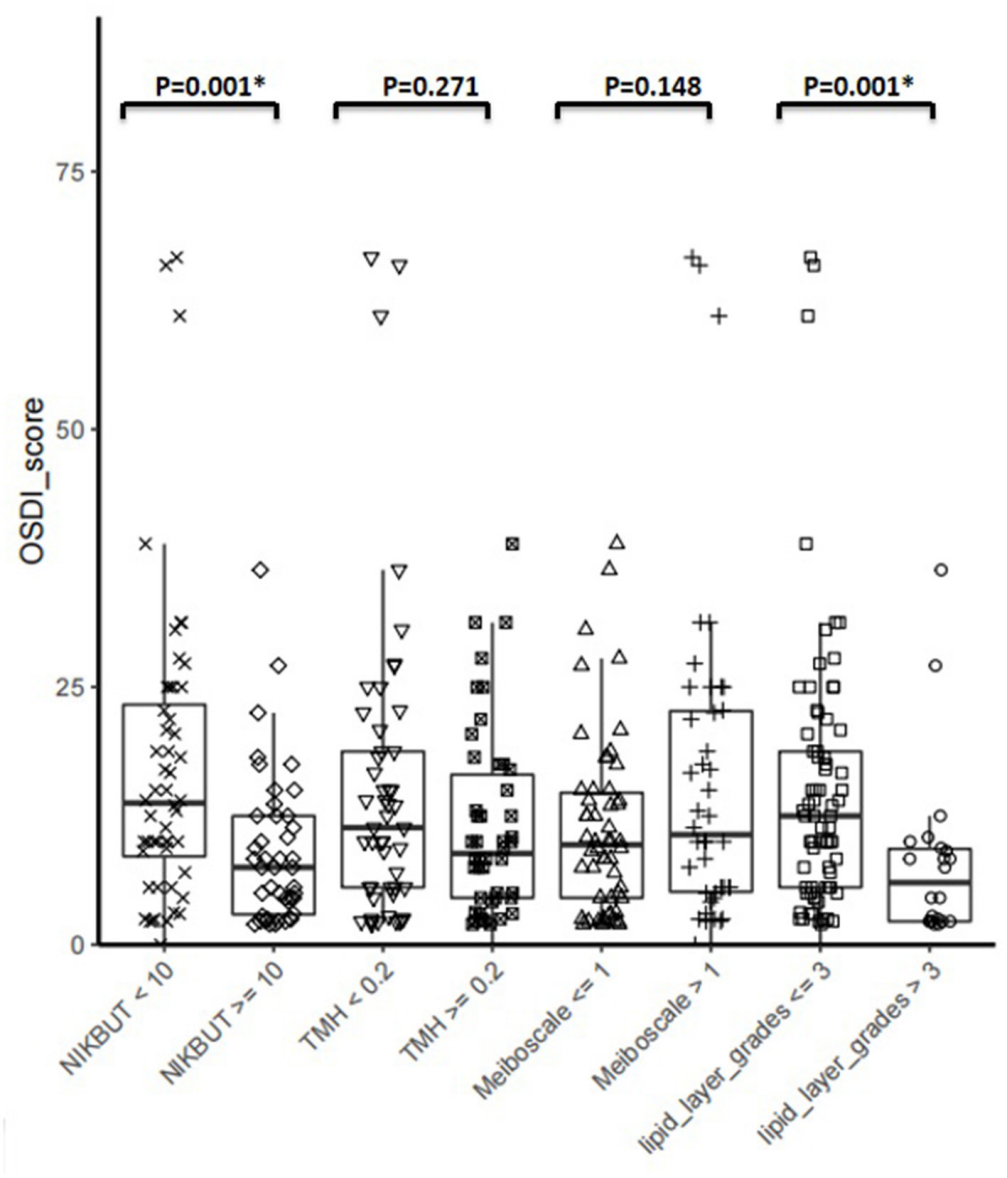
Correlation between DED symptoms and Signs. SLE patients were stratified according to published cut-off values for DED diagnostic tools using in this study, and compared their OSDI. Asterisks denote statistically significant difference (*P* < 0.05).

SLE patients were stratified according to published cut-off values for DED diagnostic tools and compared their OSDI. The results showed that patients diagnosed with DED using NIKBUT < 10 S or tear film lipid layer grade ≤3 had a statistically higher OSDI than patients who did not meet the criteria (*P* < 0.05, Figure 5).

### Difference Between Demographic and Clinical Variables in SLE Patients With and Without DED

Next, we sought to identify the possibility that confounding factors leading to the high risk of DED, particularly tobacco use, immunosuppressive drug use, and other immune-related clinical factors. According to the diagnostic criteria of DED, patients with SLE were divided into DED group and non-DED group. Table 2 describes the demographic and clinical variables of SLE patients with and without DED. Compared to non-DED patients, DED patients were older (*p* < 0.05) and had higher SLEDAI (*P* < 0.05). However, there were no significant differences between two groups in smoking, alcohol use, corticosteroids use, hydroxychloroquine use, hypertension, diabetes, CRP, C3, and ESR (all *P* > 0.05).

**Table 2:** Difference between demographic and clinical variables in SLE patients with and without DED.

**Parameters**	**DED patients in SLE (*n =* 33)**	**Non- DED patients in SLE (*n =* 63)**	***P***
Alcohol use, *n*(%)	2 (6.1%)	2 (3.2%)	0.893
Tobacco use, *n*(%)	1 (3.0%)	2 (3.2%)	0.969
Corticosteriods use, *n*(%)	24 (72.7%)	43 (68.3%)	0.650
Hydroxychloroquine use, *n*(%)	20 (60.6%)	33 (52.4%)	0.441
Hypertension	3 (9.1%)	4 (6.3%)	0.624
Diabetes	3 (9.1%)	3 (4.8%)	0.698
CRP, mg/L	0.70 (0.20,1.35)	0.60 (0.20,2.10)	0.978
C3,g/L	0.86 (0.68,1.02)	0.78 (0.64,0.92)	0.151
ESR, mm/h	19.0 (13.5,34.5)	20.0 (11.0,33.0)	0.746
SLEDAI	7.0 (5.0,13.5)	4.0 (2.0,8.5)	**<0.05**
Age	42.1 ± 11.2	33.2 ± 10.5	**<0.05**

*Data is presented as mean ± SD, median and interquartile range (IQR, 25–75%), or number of participants (% of participants). SLE, Systemic Lupus Erythematosus; CRP, C-Reactive Protein; C3, complement levels; ESR, Erythrocyte Sedimentation Rate; SLEDAI, Systemic Lupus Erythematosus Disease Activity Index. The bold values means significant results*.

### Evaluation of Predictive Value of Risk Factors of DED in SLE Patients

**Figure 6:**
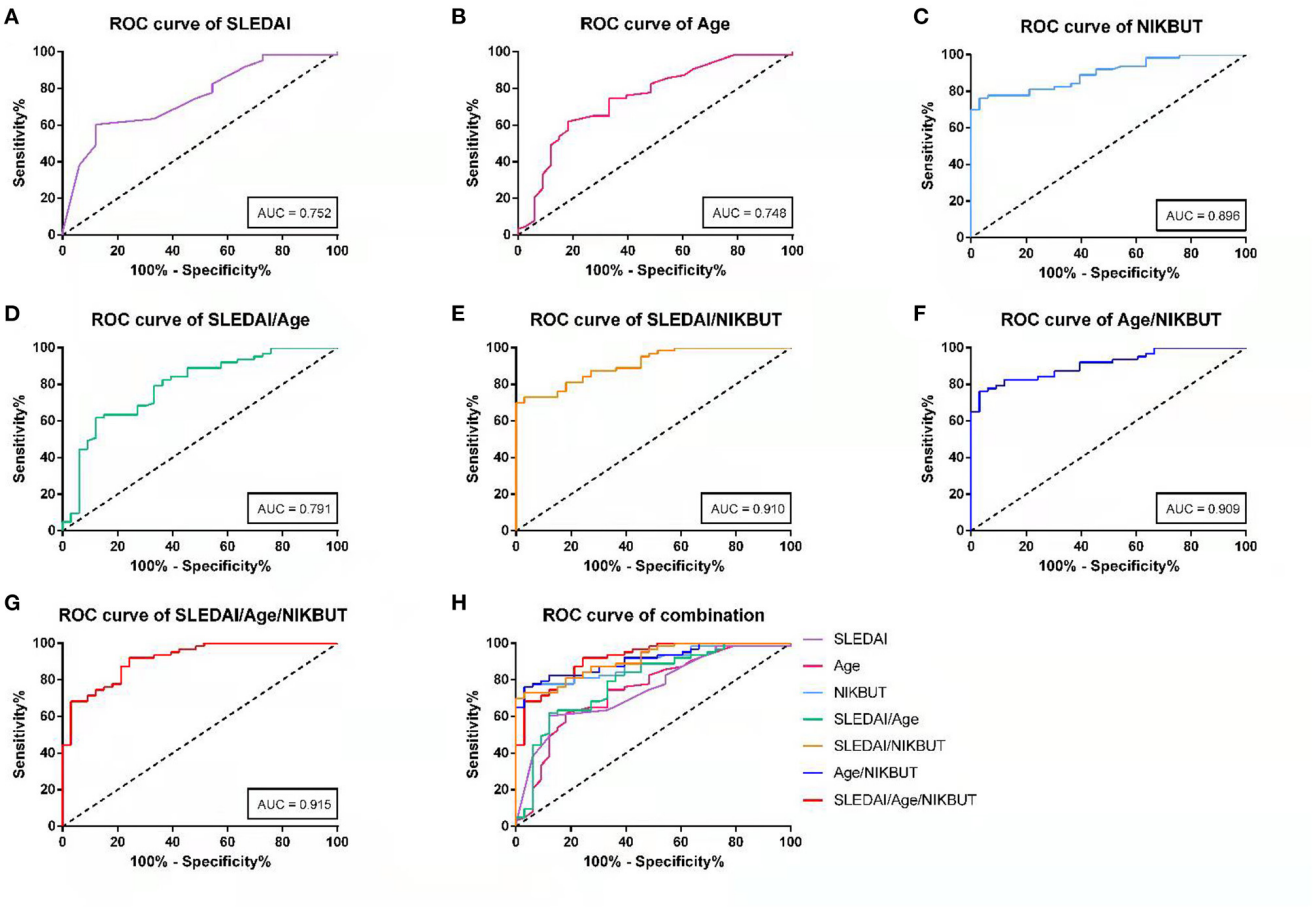
The receiver operative characteristic (ROC) curves were plotted to evaluate the predictive value of risk factors. Curves are shown for **(A)** SLEDAI, **(B)** age, **(C)** NIKBUT, **(D)** combination of SLEDAI and Age, **(E)** combination of SLEDAI and NIKBUT, **(F)** combination of Age and NIKBUT, **(G)** combination of SLEDAI, Age and NIKBUT; **(H)** comparison diagram of each curve.

ROC curves were drawed to evaluate predicted value of each risk factor. As shown in Table 3, the Youden's optimal cut off threshold of SLEDAI, age, NIKBUT for prediction was 4.5, 33.5, and 9.415, respectively. For ROC analysis, the area under curve (AUC), sensitivity and specificity of prediction were 0.752, 87.9, and 60.3% for SLEDAI; 0.748, 81.8, and 61.9% for age; and 0.896, 76.2, and 97.0% for NIKBUT, respectively. And AUC, the sensitivity, and specificity of prediction for combination of SLEDAI and age was 0.791, 87.9, and 61.9%; for SLEDAI and NIKBUT was 0.910, 97.0, and 73.0%, for age and NIKBUT was 0.909, 97.0, and 76.2%, and for three indictors was 0.915, 75.8, and 92.1% (Figure 6).

**Table 3:** Diagnosis efficiency of SLEAI, Age, NIKBUT and combo parameters for DED by ROC analysis.

**Parameters**	**Cut-off**	**Area under curve**	**Sensitivity/%**	**Specificity/%**	**Youden's index**
SLEDAI	4.5	0.752	87.9%	60.3%	0.482
Age	33.5	0.748	81.8%	61.9%	0.437
NIKBUT	9.415	0.896	76.2%	97.0%	0.732
SLEDAI+Age		0.791	87.9%	61.9%	0.498
SLEDAI+NIKBUT		0.910	97.0%	73.0%	0.700
Age+NIKBUT		0.909	97.0%	76.2%	0.732
SLEDAI+Age+NIKBUT		0.915	75.8%	92.1%	0.678

*SLEDAI, Systemic Lupus Erythematosus Disease Activity Index; NIKBUT, Non-Invasive Tear Film Breakup Time*.

## Discussion

The aim of this study was to investigate the differences in ocular surface characteristics, tear film quality, and the incidence of DED between SLE patients without SS and healthy populations. Autoimmune-related DED is not a new topic, and DED in pSS and sSS patients has been widely studied and reported (7, 11). However, there have been few studies on DED in SLE patients without SS. To our knowledge, this is the first study to evaluate the incidence, subcategories, and the potential predictors of DED in ordinary SLE patients according to the diagnostic criteria and methodology recommended by the TFOS DEWS II Diagnostic Methodology Report.

The results showed that the proportion of SLE patients who met the TFOS DEWS II DED diagnostic criteria was significantly higher than that of the control group. The current study showed that TMH was lower in SLE patients, which is consistent with the trend of previous studies of DED in patients with autoimmune diseases (23, 24). In this study, the TMH values of many SLE patients were between 0.1 and 0.2 mm, reflecting mild to moderate aqueous deficiency. However, SS patients often present with more severe aqueous deficiency (25). Our results suggested that the lacrimal gland damage in SLE patients may be less severe than that in SS patients. In this study, NIKBUT was significantly shorter in SLE group. It has been reported that NIKBUT mainly reflects the stability of tear film and decreases significantly in patients with SS (23). In addition, Poorer MG dropout and lipid layer were observed in SLE patients,which may also be involved in the occurrence of DED. MG dropout may potentially reduce the delivery of meibomian lipids to the tear film, and the damaged lipid quality can promote tear film evaporation, which might further aggravate the signs and symptoms of DED.

One of the purposes of this study was to investigate the classification of DED in SLE patients. According to TFOS DEWS II criteria, DED can be divided into three subtypes: ADDE, EDE, and mixed DED. ADDE is related to lacrimal gland dysfunction, which is more common in SS patients. EDE is mainly associated with abnormal eyelid function (e.g. MGD) (1). Considering that SLE is an autoimmune disease, we had expected more SLE patients to present with ADDE. However, in fact, the majority of SLE patients presented with mixed DED, which may imply that eyelid abnormalities are also a major cause of SLE - associated DED. Compared with healthy subjects, there was no significant difference in subcategories of DED in SLE patients.

In the past, due to the limitations of recognition and detection equipment, the diagnosis of autoimmune-related DED often focused on the destruction of the lacrimal gland and the change in tear volume, resulting in incomplete evaluation and ignoring the change in the meibomian gland and lipid layer of tear film. The meibography techniques of Keratograph 5M provide us with favorable conditions for quantifying MG dropout, which is one of the characteristic clinical manifestations of MGD (12). The results showed that SLE patients had higher MG dropout and worse tear film lipid layer compared with healthy subjects, which can cause the tear film to evaporate faster and may be an important factor in DED (26). Our study found that even if the SLEDAI was not severe, patients may still have relatively common eyelid abnormalities. SLE occurs frequently on the face and often involves the eyelids, which often show rough, red and scaling appearances. This is an interesting phenomenon. We hypothesized that eyelid involvement might lead to changes in the MG and lipid layer of tear film, contributing to the occurrence of DED. Based on the above discussion, we considered that SLE patients were more likely to present with mixed DED. It is of great significance to research the subtypes of DED in SLE patients. Identifying subtypes of DED is critical to selecting the most appropriate management strategy. This study suggested that eyelid management was an important aspect of DED treatment in SLE patients. Whether physical therapy (e.g. IPL, forceful expression of the MG) or warm compresses can be used to intervene in the progression of DED in SLE patients is the focus of our future research.

Meanwhile, there was discordance between symptoms and signs of DED in SLE patients, which has been reported in pSS (27). The results showed that SLE patients with objective signs of DED diagnosed using NIKBUT < 10 or tear film lipid layer grade ≤3 had a statistically higher OSDI than those who did not meet the criteria. However, patients with low TMH or high meiboscale were not more symptomatic. It was worth noting that some SLE patients who do not present symptoms of DED but have one or more ocular surface abnormalities may be prone to DED. This implied that more attention should be given to the possibility of DED in SLE patients and preventive management should be carried out if necessary, such as before cataract surgery for SLE patients. For these patients, surgery may increase the risk of symptomatic DED after operation (1).

This study demonstrated that compared to SLE patients without DED, DED patients were older and had higher SLEDAI. This suggested that ophthalmologists and rheumatologists should pay attention to DED in SLE patients, especially those with older age and severe disease activity. However, there were no significant differences between two groups in smoking, alcohol use, corticosteroids use, hydroxychloroquine use, hypertension, diabetes, CRP, C3, and ESR. This result suggested that immunosuppressive drugs did not increase the incidence of DED in SLE patients, which was consistent with the results of Yoon et al. (28).

Through the ROC analysis, it can be found that the AUC of SLEDAI, age, and BUT are 0.752, 0.748, and 0.896, respectively. Each of them can be used as a reference indicator for early prediction of DED in SLE patients. However, AUC of each single indicator was between 0.7 and 0.9, and the diagnostic accuracy of them for DED was moderate. When the combination of the three was selected, the AUC was the largest (>0.9), indicating that the diagnostic accuracy of DED is the highest. Therefore, the combined detection can improve the accuracy of prediction.

There were some shortcomings in this study. (1) The participants were only recruited from the First Affiliated Hospital of Anhui Medical University, so there may be restrictions due to regional restrictions. (2) Due to patient compliance, we did not include the very severe SLE, which might affect the analysis of the correlation between ocular surface parameters and SLEDAI. (3) In the future research, we need to add other DED diagnostic tools (e.g., corneal staining, and lid margin staining) to improve the accuracy of DED diagnosis.

In conclusion, the results showed that SLE patients without SS had a higher risk of developing DED than healthy populations, and tended to present with mixed DED. The poorer meibomian gland function in SLE patients may potentially contribute to the development of DED. The combined indicators of SLEDAI, age and NIKBUT showed a high accuracy for the diagonosis of DED in SLE patients.

## Data Availability Statement

The original contributions presented in the study are included in the article/supplementary material, further inquiries can be directed to the corresponding author/s.

## Ethics Statement

The present study was approved by the Ethics Committee of the First Affiliated Hospital of Anhui Medical University. Informed consent was obtained from all participants. Written informed consent to participate in this study was provided by the participants' legal guardian/next of kin.

## Author Contributions

ZG and RL: study concept and design. ZG, AZ, and QL: collecting and analyzing the data. QL and AZ: statistical expertise. ZG: writing the manuscript. RL and ZS: technical or material support, supervision, and critical revision of the manuscript. All authors contributed to the article and approved the submitted version.

## Funding

This research was supported by the National Natural Science Foundation of China (81871296), Clinical Science Foundation of Anhui Medical University (2021xkj158), and Natural Science Foundation of Universities in Anhui Province (KJ2021A0298).

## Conflict of Interest

The authors declare that the research was conducted in the absence of any commercial or financial relationships that could be construed as a potential conflict of interest.

## Publisher's Note

All claims expressed in this article are solely those of the authors and do not necessarily represent those of their affiliated organizations, or those of the publisher, the editors and the reviewers. Any product that may be evaluated in this article, or claim that may be made by its manufacturer, is not guaranteed or endorsed by the publisher.

## References

[1] Craig JP Nichols KK Akpek EK Caffery B Dua HS Joo CK. TFOS DEWS II definition and classification report. Ocul Surf. (2017) 15:276–83. 10.1016/j.jtos.2017.05.008 28736335

[2] Stapleton F Alves M Bunya VY Jalbert I Lekhanont K Malet F. TFOS DEWS II epidemiology report. Ocul Surf. (2017) 15:334–65. 10.1016/j.jtos.2017.05.003 28736337

[3] Bron AJ de Paiva CS Chauhan SK Bonini S Gabison EE Jain S. TFOS DEWS II pathophysiology report. Ocul Surf. (2017) 15:438–510. 10.1016/j.jtos.2017.05.011 28736340

[4] Massingale ML Li X Vallabhajosyula M Chen D Wei Y Asbell PA. Analysis of inflammatory cytokines in the tears of dry eye patients. Cornea. (2009) 28:1023–7. 10.1097/ICO.0b013e3181a16578 19724208

[5] Barone F Colafrancesco S. Sjogren's syndrome: from pathogenesis to novel therapeutic targets. Clin Exp Rheumatol. (2016) 34:58–62. Available online at: https://www.clinexprheumatol.org/article.asp?a=10894 27586806

[6] Godin MR Stinnett SS Gupta PK. Outcomes of thermal pulsation treatment for dry eye syndrome in patients with sjogren disease. Cornea. (2018) 37:1155–8. 10.1097/ICO.0000000000001621 29708939

[7] Moscovici BK Holzchuh R Sakassegawa-Naves FE Hoshino-Ruiz DR Albers MB Santo RM. Treatment of Sjogren's syndrome dry eye using 0.03% tacrolimus eye drop: Prospective double-blind randomized study. Cont Lens Anterior Eye. (2015) 38:373–8. 10.1016/j.clae.2015.04.004 25956572

[8] Ogawa Y. Sjogren's syndrome, non-sjogren's syndrome, and graft-versus-host disease related dry eye. Invest Ophthalmol Vis Sci. (2018) 59:DES71–DES9. 10.1167/iovs.17-23750 30481809

[9] Lee SY Han SJ Nam SM Yoon SC Ahn JM Kim TI. Analysis of tear cytokines and clinical correlations in Sjogren syndrome dry eye patients and non-Sjogren syndrome dry eye patients. Am J Ophthalmol. (2013) 156:247–53 e1. 10.1016/j.ajo.2013.04.003 23752063

[10] Yurkovich M Vostretsova K Chen W Avina-Zubieta JA. Overall and cause-specific mortality in patients with systemic lupus erythematosus: a meta-analysis of observational studies. Arthritis Care Res. (2014) 66:608–16. 10.1002/acr.22173 24106157

[11] Gilboe IM Kvien TK Uhlig T Husby G. Sicca symptoms and secondary Sjogren's syndrome in systemic lupus erythematosus: comparison with rheumatoid arthritis and correlation with disease variables. Ann Rheum Dis. (2001) 60:1103–9. 10.1136/ard.60.12.1103 11709451 PMC1753445

[12] Wolffsohn JS Arita R Chalmers R Djalilian A Dogru M Dumbleton K. TFOS DEWS II diagnostic methodology report. Ocul Surf. (2017) 15:539–74. 10.1016/j.jtos.2017.05.001 28736342

[13] Abdelfattah NS Dastiridou A Sadda SR Lee OL. Noninvasive imaging of tear film dynamics in eyes with ocular surface disease. Cornea. (2015) 34:S48–52. 10.1097/ICO.0000000000000570 26226477

[14] Hochberg MC. Updating the American College of Rheumatology revised criteria for the classification of systemic lupus erythematosus. Arthritis Rheum. (1997) 40:1725. 10.1002/art.1780400928 9324032

[15] Vitali C Bombardieri S Jonsson R Moutsopoulos HM Alexander EL Carsons SE. Classification criteria for Sjogren's syndrome: a revised version of the European criteria proposed by the American-European Consensus Group. Ann Rheum Dis. (2002) 61:554–8. 10.1136/ard.61.6.554 12006334 PMC1754137

[16] Schiffman RM Christianson MD Jacobsen G Hirsch JD Reis BL. Reliability and validity of the ocular surface disease index. Arch Ophthalmol. (2000) 118:615–21. 10.1001/archopht.118.5.615 10815152

[17] Guillon JP. Use of the Tearscope Plus and attachments in the routine examination of the marginal dry eye contact lens patient. Adv Exp Med Biol. (1998) 438:859–67. 10.1007/978-1-4615-5359-5_121 9634979

[18] Pult H Riede-Pult B. Comparison of subjective grading and objective assessment in meibography. Cont Lens Anterior Eye. (2013) 36:22–7. 10.1016/j.clae.2012.10.074 23108007

[19] Craig JP Wang MT Kim D Lee JM. Exploring the predisposition of the asian eye to development of dry eye. Ocul Surf. (2016) 14:385–92. 10.1016/j.jtos.2016.03.002 27143647

[20] Wang MTM Xue AL Craig JP. Screening utility of a rapid non-invasive dry eye assessment algorithm. Cont Lens Anterior Eye. (2019) 42:497–501. 10.1016/j.clae.2018.11.009 30473321

[21] Wolffsohn JS Wang MTM Vidal-Rohr M Menduni F Dhallu S Ipek T. Demographic and lifestyle risk factors of dry eye disease subtypes: a cross-sectional study. Ocul Surf. (2021) 21:58–63. 10.1016/j.jtos.2021.05.001 33965652

[22] Gladman DD Ibanez D Urowitz MB. Systemic lupus erythematosus disease activity index 2000. J Rheumatol. (2002) 29:288–91. 11838846

[23] Wang Y Qin Q Liu B Fu Y Lin L Huang X. Clinical analysis: aqueous-deficient and meibomian gland dysfunction in patients with primary sjogren's syndrome. Front Med. (2019) 6:291. 10.3389/fmed.2019.00291 31921869 PMC6914862

[24] Menzies KL Srinivasan S Prokopich CL Jones L. Infrared imaging of meibomian glands and evaluation of the lipid layer in Sjogren's syndrome patients and nondry eye controls. Invest Ophthalmol Vis Sci. (2015) 56:836–41. 10.1167/iovs.14-13864 25574045

[25] Koh S Ikeda C Watanabe S Oie Y Soma T Watanabe H. Effect of non-invasive tear stability assessment on tear meniscus height. Acta Ophthalmol. (2015) 93:e135–9. 10.1111/aos.12516 25308575

[26] Willcox MDP Argueso P Georgiev GA Holopainen JM Laurie GW Millar TJ. TFOS DEWS II tear film report. Ocul Surf. (2017) 15:366–403. 10.1016/j.jtos.2017.03.006 28736338 PMC6035753

[27] Vehof J Sillevis Smitt-Kamminga N Nibourg SA Hammond CJ. Predictors of discordance between symptoms and signs in dry eye disease. Ophthalmology. (2017) 124:280–6. 10.1016/j.ophtha.2016.11.008 28024826

[28] Yoon CH Lee HJ Lee EY Lee EB Lee WW Kim MK. Effect of hydroxychloroquine treatment on dry eyes in subjects with primary sjogren's syndrome: a double-blind randomized control study. J Korean Med Sci. (2016) 31:1127–35. 10.3346/jkms.2016.31.7.1127 27366013 PMC4901007

